# Knowledge and Outcome Measure of HbA1c Testing in Asian Indian Patients with Type 2 Diabetes from a Tertiary Care Center

**DOI:** 10.4103/0970-0218.66858

**Published:** 2010-04

**Authors:** Satyavani Kumpatla, Srikanth Medempudi, Deepa Manoharan, Vijay Viswanathan

**Affiliations:** Diabetes Research Centre and M.V. Hospital for Diabetes (WHO Collaborating Centre for Research, Education and Training in Diabetes), No. 5, Main Road, Royapuram, Chennai, Tamil Nadu, India

**Keywords:** Asian Indians, awareness, HbA1c, type 2 diabetes

## Abstract

**Aim:**

HbA1c test is considered to be the reliable measure for evaluating long-term glycemic control in type 2 diabetes. The purpose of this study was to evaluate whether knowledge about HbA1c test is associated with a better glycemic control.

**Materials and Methods:**

We conducted a cross-sectional survey of 480 (M:F; 287:193) adults with type 2 diabetes attending a tertiary care center during a period of four months. Baseline demographic and clinical data of all the subjects was obtained. Subject’s knowledge about HbA1c test and their target goal was assessed using a questionnaire. Recent HbA1c results were obtained from medical records.

**Results:**

Seventy four per cent of the subjects had awareness about HbA1c test and about 43% of those who knew HbA1c test also knew their target goal. 33% remember their last HbA1c result. The mean A1C of Group A was significantly lower when compared with Group B (8.1 ± 1.7 *vs* 9.2 ± 1.9, P<0.0001). Group C had lower A1C levels compared to Group D (7.7 ± 1.4 *vs* 8.5 ± 1.9, p<0.0001). Patients who kept their HbA1c less than 7% were significantly higher in Group C than in Group D. (37.8 vs 12.7%, p<0.00001). Subjects had good glycemic control with increasing levels of awareness about HbA1c.

**Conclusion:**

Majority of the diabetic patients who attended the tertiary care center for diabetes care knew HbA1c test and half of them were aware about their target goal. Awareness about HbA1c had a positive impact on maintenance of better glycemic control.

## Introduction

Type 2 diabetes is a major health problem and an escalating prevalence of type 2 diabetes is seen in developing countries such as India.(1,2) The patients with type 2 diabetes are at high risk of developing vascular complications. Several studies have reported that improved glycemic control can reduce the development and/or progression of diabetic complications.(3,4) The glycosylated hemoglobin (A1C) test has been the most widely accepted, reliable outcome measure for evaluating long-term glycemic control and this test provides an index of average blood glucose level during the past 2-3 months.(5,7) Maintenance of A1C levels as close as possible to the near normal range results in considerable reduction in long-term complications of diabetes.(3,4) This test provides important feedback to both health care professionals and patients. Patient’s understanding of HbA1c and its target goal will definitely have a positive impact on long-term health.(8)

American Diabetes Association recommends diabetic patients to be aware of their target and actual HbA1c value.(9) Several studies have been conducted on the effectiveness of diabetes education(10–12) and all these studies have clearly shown a beneficial effect of education and motivation on diabetes control and reduction of complications, but there is paucity of data on cross-sectional studies.

Despite the efforts by many organizations in raising public awareness on the role of HbA1c in the development of diabetes-related complications, most patients with the disease have never heard of the term HbA1c and do not know their HbA1c levels and target goal. Many studies underscore the opportunities missed by physicians for providing diabetes education and counseling aimed at optimizing glycemic control.(13–16) Among large managed care organizations such as Health Maintenance Organization (HMO) and Place of Service (POS) that provide health care in return for a predetermined monthly fee and coordinate care through a defined network of physicians and hospitals where 92% of the patients perform self-monitoring of blood glucose, but less than one-third have heard of the HbA1c test and very few among them know their goals.(14,15) Findings from a study conducted in the United States showed that 66% of the patients did not know their last A1c results, with only 25% able to accurately report the value.(17)

People who are aware of their health goals and believe that these goals are within their control have improved outcomes and they get engaged in self-care behaviors, including exercise and weight loss programs.(18–20) The aim of this study was to assess whether knowledge about HbA1c test and its target goal is associated with a better glycemic control among type 2 diabetic patients.

## Materials and Methods

We conducted a cross-sectional study and the study subjects (*n*=480, M:F; 287:193) were selected from an outpatient department of a tertiary care center in India during a period of four months in the year 2008. Diabetic patients of all socio-economic strata attended the center for routine management of diabetes. Consecutive type 2 diabetic patients who attended the center as their primary location for diabetes care and who had been diagnosed with diabetes for a minimum of one year or longer were included in this study. It has been a clinical practice goal in the center to measure HbA1c every 3 months and to educate patients about the test, their result and their target goal as recommended by American Diabetes Association.(9,21) Patients with a history of renal insufficiency with a creatinine level >1.5mg/dl, pregnant women and patients using insulin pumps, patients who received a blood transfusion within the past 30 days and those with known underlying illness, such as malignancy, hemoglobinopathies were excluded. All the eligible patients were informed that they were entering an educational study designed to assess their knowledge and the impact of HbA1c awareness on overall glycemic control. Informed consent was obtained from patients who agreed to participate in the study. Ethics committee of the institution approved the study. The details of baseline demographic data, age, sex, location, monthly income, educational status, duration of diabetes as well as antidiabetic medications were recorded.

A researcher-administered questionnaire was employed to test respondent’s knowledge about HbA1c test and their target goal. Each respondent is presented with three questions in the same order. The choice of answers to the questions is open ended. Respondents were asked, “What does HbA1c test mean?” (Respondents were classified as having accurate awareness about the test (group A) if they answered it as overall glycemic control test or 2-3 months blood sugar control test. Respondents were coded as unaware of the test (group B) if they answered wrongly or if responded, “I don’t know”. Respondents who were aware of the test were then asked “What is your HbA1c goal? (We classified the respondents as ‘aware and goal known’ (group C) if they mentioned their target goal as less than 7%. Respondents were coded as ‘goal not known’ (group D) if they answered wrongly or if responded, “I don’t know”. Respondents who were aware of their goal were then asked, “What is your last HbA1c result?" (We classified respondents as knowing their HbA1c value if their actual test result was within 0.5 percentage points of the lower or upper boundary of the mentioned value. For example, if the respondents reported that their HbA1c was 7, they were grouped as knowing their HbA1c (group E) if their recorded HbA1c was within 6.5-7.5. Respondents were coded as not knowing their value if their estimate differed by >0.5% or if responded, “I don’t know”.

We reviewed medical records and/or laboratory data to document respondent’s most recent HbA1c results taken before the survey. If respondents had no documented HbA1c result, we recorded this value as ‘no result’.

## Statistical analysis

Statistical analyses were performed with the SPSS version 10 package. Descriptive statistics were computed with mean and SD for continuous measurements. Group comparisons were done by Student’s ‘t’ test or by Chi-square test as relevant. *P* <0.05 was regarded as statistically significant.

## Results

**Figure 1:**
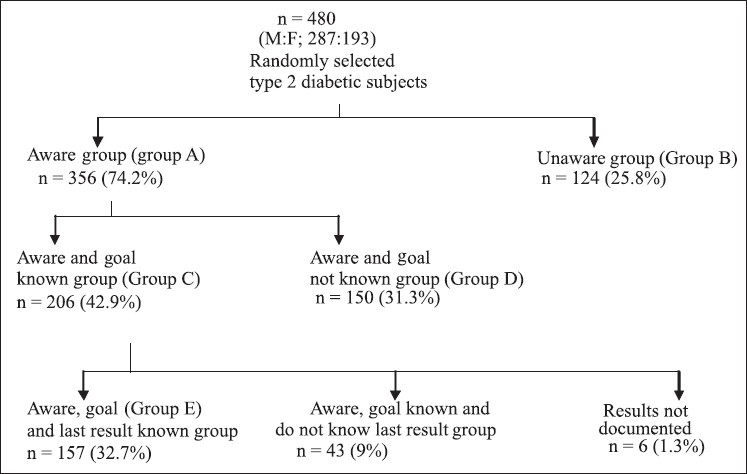
Shows the details of knowledge about HbA1c test among study subjects

The mean age of study subjects was 53.5 ± 11.4 years and mean duration of diabetes was 9.3 ± 7.2 years. More than 65% of the study subjects are treated with OHA and 4% were on insulin alone and 25% on combination therapy. Figure 1 shows the details of knowledge about HbA1c test among study subjects. Question 1 assessed subject’s awareness about HbA1c test. 74% of the subjects know about HbA1c test and 26% were unaware of the HbA1c test. Question 2 assessed subject’s awareness about their A1C goal. About 43% of those who know about HbA1c know their goal also. 31 % are aware about HbA1c test but they do not know their goal. Question 3 assessed remembrance of last HbA1c result among subjects who are aware and know their goal. 33% of the subjects remember their last HbA1c result and about 9% do not know their last A1C result and 6 subjects do not have documented last HbA1c result.

Table 1 shows the comparison of demographic and clinical details between group A and group B subjects. Mean age and duration of diabetes were similar in both the groups. Mean HbA1c % was significantly lower in group A compared to group B (8.1 ± 1.7 *vs* 9.2 ± 1.9 %, *P*<0.0001). Proportion of subjects with higher education was more in group A than in group B. Majority of the study subjects belong to urban location and middle income category.

**Table 1:** Comparison of demographic and clinical details between Group A and Group B subjects

	Group A	Group B	*P* value
N, M:F	356 (211:145)	124 (76:48)	
Values are mean ± SD			
Age (years)	53 ± 11.5	55 ± 10	0.086
Duration of diabetes (years)	9.2 ± 7.3	10.6 ± 7.2	0.066
Mean HbA1c (%)	8.1 ± 1.7	9.2 ± 1.9	<0.0001
Values are n (%)			
Education			
Illiterate	16 (4.5)	23 (18.5)	<0.0001
School/high School	199 (55.9)	77 (62.1)	0.27
Graduate/post graduate	141 (39.6)	24 (19.4)	<0.0001
Location			
Urban	320 (89.8)	118 (95.2)	NS
Rural	36 (10.1)	6 (4.8)	NS
Monthly income (Indian rupees)			
<10000	10 (2.8)	11 (8.9)	0.009
10000-25000	300 (84.5)	105 (84.7)	NS
>25000	46 (12.9)	8 (6.5)	NS
Treatment			
OHA	254 (71.3)	82 (66.1)	NS
Insulin	14 (3.9)	5 (4.0)	NS
OHA + Insulin	88 (24.7)	37 (29.8)	NS

NS – Non Significant; Group A: Aware Group; Group B: Unaware Group

**Figure 2:**
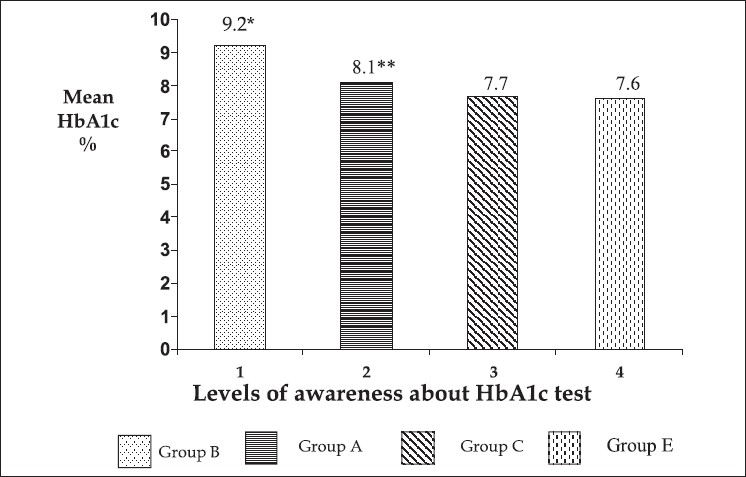
Shows the mean HbA1c (%) at different levels of awareness about the HbA1c test. *1*vs* 2; *P*<0.0001; ** 2 *vs* 3; *P*=0.004; Group A: Aware group; Group B: Unaware group; Group C: Aware and goal known group; Group D: Aware and goal not known group; Group E: Aware, goal and last result known group

Table 2 shows the comparison of details of HbA1c in group C versus group D subjects. Group C had significantly lower HbA1c levels than group D (7.7 ± 1.4 *vs* 8.5 ± 1.9, *P*< 0.0001). 35% of those who knew their goal were able to maintain their HbA1c % less than 7. Education levels differed between group C and group D. Figure 2 shows the mean HbA1c % at different levels of awareness about HbA1c test. Maintenance of good glycemic control was seen with increasing levels of awareness about HbA1c test.

**Table 2:** Comparison of details of HbA1c in Group C versus Group D subjects

	Group C	Group D	*P* value
N, (M:F)	206 (127:79)	150 (84:66)	
Values are mean ± SD			
Age (years)	52 ± 11.8	54.5 ± 10.8	0.041
Duration of diabetes (years)	9.3 ± 7.6	8.9 ± 6.7	0.606
Values are n (%)			
Education			
Illiterate	3(1.5)	13(8.7)	0.003
School/high School	98(47.6)	101(67.3)	<0.0001
Graduate/post graduate	105(51)	36(24)	<0.0001
Location			
Urban	187(90.8)	133(88.7)	NS
Rural	19(9.2)	17(11.3)	NS
Monthly income (Indian rupees)			
<10000	8(3.9)	5(3.3)	NS
10000-25000	196(95)	144(96)	NS
>25000	2(0.9)	1(0.7)	NS
Mean HbA1c (%)	7.7 ± 1.4	8.5 ± 1.9	<0.00001
HbA1c <7%, n (%)	78 (37.8)	19 (12.7)	<0.00001

NS – Non significant; Group C: Aware and goal known group; Group D: Aware and goal not known group

## Discussion

The present study showed that a large number of type 2 diabetic patients knew about HbA1c test and nearly 43% are aware about their goal also. There was a significant difference in mean HbA1c levels between aware and unaware groups with age, duration of diabetes and treatment modality matched subjects. Subjects who were aware of HbA1c test and their goal had a better glycemic control compared to subjects who were not aware of HbA1c test. Patients who were not aware of the HbA1c test were educated concerning the meaning of the test and explained about their target goal. A cross-sectional study from United States (17) examined the relationship between patient’s knowledge of their recent HbA1c value and self-management of diabetes. It was reported that only 25% were able to accurately report the HbA1c values. Another study on type 1 diabetic patients concluded that more than 80% of the studied subjects knew their last HbA1c value (22)and they had high perceived knowledge about HbA1c testing, whereas in our study about 33% knew their last HbA1c results.

In our study, majority of the subjects had knowledge about HbA1c test and this might be because of longer duration of diabetes. Approximately half of the subjects were aware about their HbA1c goal and had a better glycemic control which implies goal oriented motivation is necessary for patients.

Mean HbA1c levels were high in subjects who were not aware of the test compared to aware group. Subjects who were aware and knew their goal also had significantly lower HbA1c levels than aware group. No significant difference was noted in the HbA1c values among the subjects who were aware and knew their goal in comparison with the subjects who were aware, goal and last result known group. The results showed that knowledge and awareness about HbA1c test and its target goal contributed to better glycemic control.

Although we demonstrated that patients empowered with HbA1c awareness and target goals could potentially have a significant impact on short-term HbA1c outcomes, further study is necessary to determine the long-term implications of HbA1c awareness, in terms of diabetes complications and outcomes. These strategies must be combined with other behavioral strategies to motivate and help patients effectively manage their diabetes.

Clinicians and diabetes educators should not only educate the patients about HbA1c test but also teach them about their target goals. Knowing about the overall glycemic control test, their goal and last HbA1c result motivate patients to effectively manage their diabetes, as well as it positively reinforces those patients who are already effectively managing their diabetes.

In conclusion, a majority of the diabetic patients knew about HbA1c test and approximately half of the patients were aware about their target goal. Knowledge and awareness about HbA1c test and their target goal had a positive impact on maintenance of better glycemic control in terms of HbA1c levels.
